# Noncanonical Splice Site Disruption: +4 Intronic Variant in Phosphate-Regulating Endopeptidase Homolog, X-linked (PHEX Gene) Supported by In Silico Analysis in X-linked Hypophosphatemic Rickets

**DOI:** 10.7759/cureus.114812

**Published:** 2026-08-19

**Authors:** Carlos F Panqueba Arias, Ingry K Rojas Rodriguez, Rossi I Quero, Cielo C Baez

**Affiliations:** 1 Pediatrics, Erasmo Meoz University Hospital, Cúcuta, COL; 2 Pediatric Endocrinology, Private Practice, Cúcuta, COL

**Keywords:** hypophosphatemia, intronic variant, phex, splicing, x-linked hypophosphatemic rickets

## Abstract

X-linked hypophosphatemic rickets (XLH) is the most common inherited cause of renal phosphate wasting. It is typically caused by pathogenic variants in the phosphate-regulating endopeptidase homolog, X-linked (PHEX gene), which lead to increased levels of fibroblast growth factor 23 (FGF23). However, noncanonical intronic variants represent a significant diagnostic challenge, as they may not be detected by conventional genetic studies and require interpretation supported by bioinformatic tools. We present the case of a pediatric patient with postnatal short stature and progressive genu varum. Biochemical studies revealed hypophosphatemia with reduced tubular reabsorption of phosphate, while calcium, parathyroid hormone (PTH), and vitamin D levels were within normal ranges. Radiological findings were consistent with rickets. Initial clinical exome sequencing was negative. Subsequently, a targeted gene panel identified a previously unreported intronic variant in the PHEX gene (c.1768+4dup; chrX:22,219,105 T>TA). In silico analysis using SpliceAI demonstrated a high probability of donor splice site loss (DS_DL = 0.80), while Pangolin predicted a splice-disrupting effect (score = 0.72); collectively, these findings support a deleterious effect on mRNA splicing. This case highlights the importance of integrating clinical, biochemical, genetic, and computational data for the interpretation of noncanonical intronic variants, expanding the mutational spectrum described for PHEX in XLH.

## Introduction

Hypophosphatemic rickets comprises a heterogeneous group of disorders characterized by renal phosphate wasting, impaired bone mineralization, and growth retardation, with clinical manifestations ranging from mild abnormalities to significant skeletal deformities [1,2]. The most common form is X-linked hypophosphatemic rickets (XLH) (OMIM #307800), accounting for more than 80% of inherited hypophosphatemia cases, with an approximate incidence of one in 20,000 live births [3]. Historically, this condition was described as “vitamin D-resistant rickets” by Albright, Butler, and Bloomberg in 1937, later redefined as “familial hypophosphatemia” in 1958, and subsequently associated with variants in the PHEX gene in 1995, with the central role of fibroblast growth factor 23 (FGF23) as a key regulator of mineral homeostasis established in 2000 [4].

XLH is caused by pathogenic variants in the phosphate-regulating endopeptidase homolog, X-linked (PHEX gene) (OMIM #300550), which is located on the X chromosome [5]. Loss of function of this gene leads to an increase in FGF23, which plays a fundamental role in phosphate homeostasis by reducing renal phosphate reabsorption through suppression of the type IIa sodium-phosphate (NaPi-IIa) and type IIc sodium-phosphate (NaPi-IIc) cotransporters, as well as by decreasing the synthesis of 1,25-dihydroxyvitamin D, thereby impairing intestinal phosphate absorption and resulting in chronic hypophosphatemia, the main biochemical feature of XLH [2].

To date, more than 640 variants in the PHEX gene have been described in the Human Gene Mutation Database (HGMD), although only a limited proportion have undergone functional characterization [5]. While variants located at canonical splice sites are typically considered pathogenic, intronic variants outside the ±1/±2 positions pose a significant diagnostic challenge [6,7]. Their interpretation requires careful integration of clinical, biochemical, and genetic findings, complemented by in silico tools capable of estimating their functional impact.

## Case presentation

We present the case of a four-year-old male patient from Cúcuta, Colombia, who initially presented at 26 months of age with short stature and progressive deformities of the lower limbs, clinically evident as genu varum. His medical history was notable for parental consanguinity (second cousins), with no known family history of bone disease or metabolic disorders. Maternal height was 160 cm and paternal height was 178 cm. He has one healthy sibling and was born after an uncomplicated second pregnancy, with normal prenatal ultrasounds and no structural anomalies. He was delivered at 37.4 weeks of gestation by vaginal delivery, with a birth weight of 2595 g (-1.61 SD) and a length of 47 cm (-1.62 SD), without neonatal complications.

Psychomotor development was globally appropriate; however, a mild delay in gross motor milestones was observed, with the ability to sit at 10 months, crawl at 11 months, and walk independently at 18 months. In contrast, language development and sphincter control occurred within expected age ranges. During anthropometric follow-up, a progressive decline in linear growth was documented, with a height of 83.5 cm (-3 SD) at 39 months and 86.5 cm (-3 SD) at 49 months, associated with a growth velocity below 4 cm/year, clearly pathological. Physical examination revealed bilateral genu varum without other dysmorphic features or relevant systemic findings. 

Laboratory studies

Biochemical evaluation (Table 1) showed hypophosphatemia associated with decreased tubular phosphate reabsorption (TRP: 84%). Serum calcium, parathyroid hormone (PTH), and 25-hydroxyvitamin D levels were within normal ranges. Renal and hepatic function were preserved, with no evidence of glucosuria or proteinuria, findings suggestive of a primary disorder of renal phosphate handling. In conjunction with the clinical manifestations, this biochemical profile was highly suggestive of an FGF23-mediated disorder.

**Table 1:** Summary of laboratory findings PTH: parathyroid hormone; GGT: gamma-glutamyl transferase; ALT: alanine aminotransferase; AST: aspartate aminotransferase; TSH: thyroid-stimulating hormone; ↓ = below normal range

Parameter	Result	Reference range
Intact PTH	65 pg/mL	15-65 pg/mL
25-hydroxyvitamin D	82.2 ng/mL	30-80 ng/mL
Alkaline phosphatase	215 U/L	<350 IU/L
Serum creatinine	0.30 mg/dL	Normal for age
Serum calcium	10 mg/dL	9.4-10.8 mg/dl
Urinary calcium (24 h)	98 mg/24h	<300 mg/24h
Urine calcium/creatinine ratio	0.01 mg/mg	<0.2 mg/mg
Serum phosphorus	4.0 mg/dL ↓	4.5-6.2 mg/dl
Tubular reabsorption of phosphate	84% ↓	>85%
Serum glucose	84 mg/dL	70-100 mg/dl
Albumin	4.2 g/dL	3.5-5 g/dl
GGT	12 IU/L	3-22 IU/L
ALT	8.8 IU/L	<40 IU/L
AST	12.5 IU/L	<40 IU/L
TSH	2.8 mUI/L	0.5-5.4 mUI/L
Free thyroxine (free T4)	1.4 ng/dl	1.1-2 ng/dl
Hemoglobin	12.5 g/dL	12.5-14.5 g/dL
Urine glucose	Negative	Negative
Urine proteins	Negative	Negative

Imaging findings

**Figure 1:**
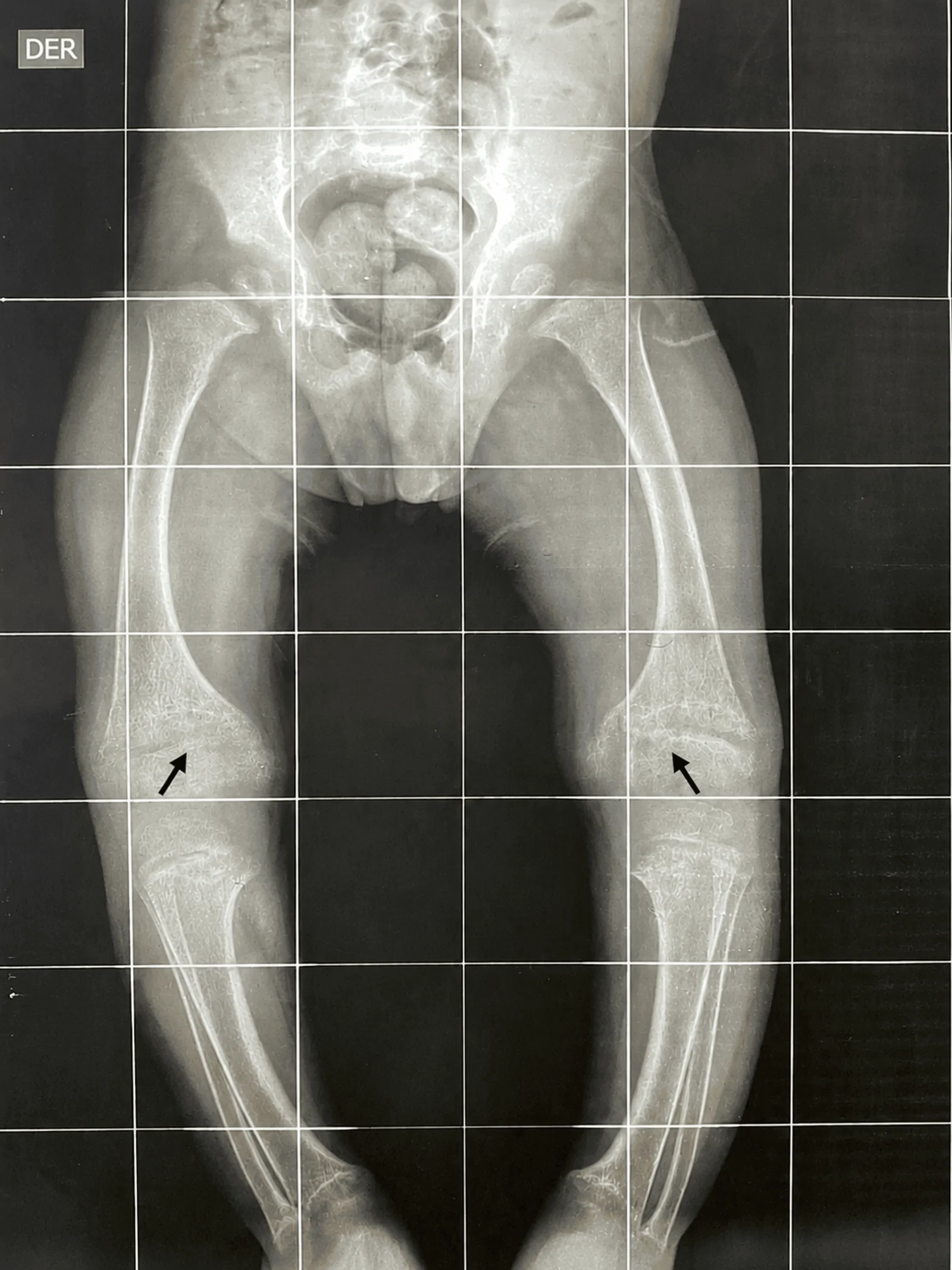
Lower limb radiograph DER: right Anteroposterior radiograph of the lower limbs demonstrating bilateral genu varum, with arrows indicating metaphyseal widening and irregular, frayed metaphyseal margins, characteristic findings of active rickets. The radiological severity corresponds to a Thacher score of 2.0 and is consistent with impaired bone mineralization secondary to renal phosphate wasting

**Figure 2:**
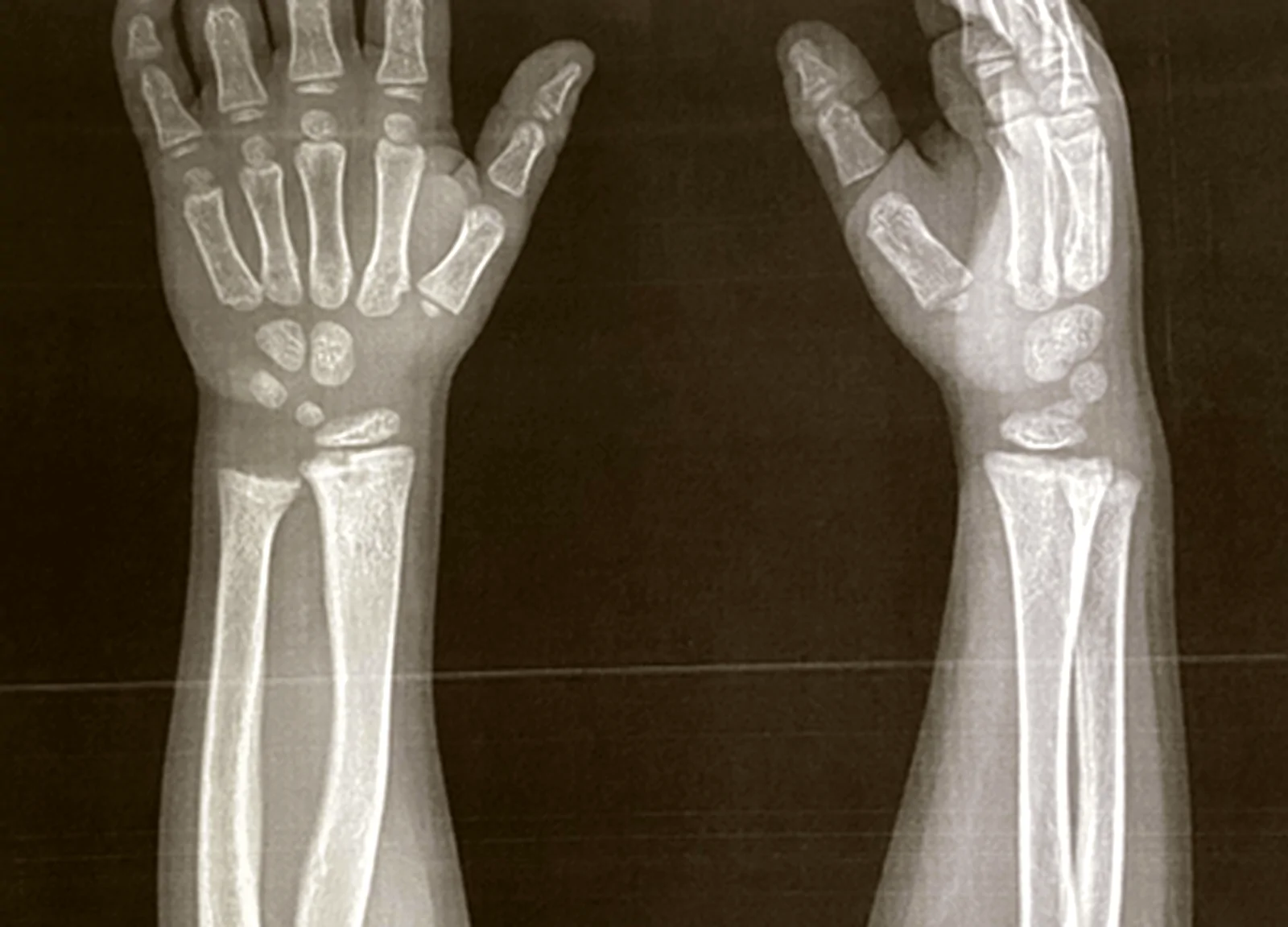
Wrist radiograph Anteroposterior and lateral radiographs of the wrist demonstrating distal metaphyseal widening of the radius and ulna, with fraying, characteristic findings of active rickets. No significant delay in bone age is observed

Radiological studies demonstrated findings characteristic of active rickets. Anteroposterior radiographs of the lower limbs showed bilateral genu varum, with metaphyseal widening and irregular, frayed metaphyseal margins, corresponding to a score of 2.0 according to the Thacher radiological classification (Figure 1) [8]. Similarly, wrist radiographs revealed distal metaphyseal widening of the radius and ulna, with changes consistent with active rickets and a score of 1 based on the Thacher classification (Figure 2). No significant delay in bone maturation was observed, suggesting a predominant defect in bone mineralization rather than overall skeletal development.

Genetic studies

Initial clinical exome sequencing did not identify pathogenic or likely pathogenic variants. Therefore, molecular testing was performed using a targeted next-generation sequencing (NGS) gene panel for hypophosphatemic rickets and related disorders, including the following genes: ALPL, CLCN5, CTNS, CYP27B1, CYP2R1, DMP1, ENPP1, FAH, FAM20C, FGF23, FGFR1, GNAS, KL, OCRL, PHEX, SLC34A1, SLC34A3, and VDR.

Genomic DNA was prepared and enriched for targeted regions, including coding exons and adjacent intronic sequences, using a customized probe-based capture system (Twist Bioscience, version 3). Sequencing was performed on an Illumina NovaSeq 6000 platform. The obtained sequences were aligned to the human reference genome (GRCh38), and genetic variants were identified using standard bioinformatics tools. Copy number variations (CNVs) were also evaluated.

This testing identified an intronic variant in the PHEX gene (c.1768+4dup; chrX:22,219,105 T>TA), which has not been previously reported in clinical databases or the scientific literature. The variant was detected in six of 25 sequencing reads at the analyzed position, corresponding to a variant allele fraction of approximately 24%. Because of the relatively low variant allele fraction, the finding was further evaluated by Sanger sequencing as an orthogonal confirmatory method. Briefly, the genomic region containing the PHEX variant was amplified by polymerase chain reaction (PCR), followed by targeted Sanger sequencing. The variant was confirmed by Sanger sequencing, confirming the presence of PHEX c.1768+4dup. In silico analysis demonstrated a high SpliceAI score (DS_DL = 0.80), indicating a strong probability of donor splice site loss [9]. Additionally, other bioinformatic predictors were concordant with altered mRNA processing, including Pangolin (score 0.72), supporting a potentially significant functional impact of the variant (Table 2) [10]. 

**Table 2:** Characteristics of the variant identified in the PHEX gene ACMG: American College of Medical Genetics and Genomics; VUS: variant of uncertain significance; PHEX gene: phosphate-regulating endopeptidase homolog, X-linked

Feature	Description
Gene	Phosphate-regulating endopeptidase homolog, X-linked (PHEX)
Variant	c.1768+4dup
Genomic position	chrX:22,219,105 T>TA
Transcript	ENST00000379374
Population frequency	Not reported
Clinical databases	Not reported in ClinVar or gnomAD v4.1.0
Variant allele fraction	~24% (suggestive of somatic mosaicism)
In silico prediction	SpliceAI DS_DL = 0.80; Pangolin splice score = 0.72
ACMG criteria	PP3 (computational evidence supporting a deleterious effect), PM2 (absent from population databases)
Classification	VUS, with computational evidence and clinicobiochemical correlation suggesting potential clinical relevance

Although Sanger sequencing confirmed the presence of the variant, the low allelic fraction observed by NGS raises the possibility of low-level mosaicism. However, it was not possible to determine the extent or biological relevance of this mosaicism based on the available data. Therefore, the molecular finding was interpreted in the context of the clinical and biochemical phenotype, which was highly consistent with an FGF23-mediated disorder.

Evolution and management

Based on the integration of clinical, biochemical, and genetic findings, a diagnosis of XLH was established. The case was reported to the epidemiological surveillance system in Colombia, and treatment with burosumab was initiated at a dose of 10 mg every two weeks. However, treatment continuity was affected by difficulties in accessing the medication. The patient is currently under interdisciplinary follow-up with ongoing clinical, biochemical, and radiological monitoring.

## Discussion

Pathogenic variants in hemizygous or heterozygous states in the PHEX gene are associated with XLH, a disorder with a broad clinical spectrum ranging from isolated hypophosphatemia to progressive skeletal deformities, such as bowing of the lower limbs [11]. Clinical signs usually become evident during the first two years of life, coinciding with the onset of bipedal stance and the increase in mechanical load on the developing skeleton, a stage at which abnormalities in bone mineralization and progression of genu varum become more pronounced [2].

This case highlights the importance of early recognition of the clinical manifestations of XLH, including disproportionate short stature and genu varum, findings that may go unnoticed in the absence of appropriate growth monitoring. From a biochemical perspective, the combination of persistent hypophosphatemia, decreased tubular reabsorption of phosphate, normal serum calcium concentrations, and PTH levels within the normal range or slightly elevated constitutes a pattern highly suggestive of an FGF23-mediated disorder [2]. In this context, the finding of PTH levels at the upper limit of normal in our patient is consistent with the underlying disease mechanism, as increased FGF23 activity reduces the production of 1,25-dihydroxyvitamin D, which normally inhibits parathyroid cell proliferation; its deficiency may therefore promote secondary hyperparathyroidism, while additional mechanisms such as altered PTH secretion and loss of PHEX function within the parathyroid gland affecting PTH mRNA processing, have also been proposed [12]. Accordingly, it has been reported that approximately 25% of patients with XLH may develop secondary hyperparathyroidism without hypercalcemia [13], adding further complexity to the interpretation of biochemical findings.

A relevant aspect of this case was the negative exome sequencing result, followed by the identification of the variant through a targeted next-generation sequencing panel. This finding underscores the limitations of exome sequencing compared to targeted panels, particularly when variants are located in regions with insufficient coverage or in noncanonical splicing site positions, circumstances that may lead to false-negative results [14]. In contrast, targeted gene panels often provide greater depth and uniformity of coverage in genes associated with a specific phenotype, thereby increasing the sensitivity for variant detection [15].

In this context, the c.1768+4dup variant in the PHEX gene is located at position +4 of the donor splice site, outside the canonical (+1/+2) positions, suggesting a potential effect on messenger RNA processing [6,7]. Although this variant has not been previously reported in the literature or databases such as ClinVar, the presence of pathogenic variants in adjacent regions supports the functional relevance of this site for proper RNA processing and protein integrity [6,16].

Although variants located outside canonical splice site positions have traditionally been more difficult to interpret, growing evidence indicates that alterations in nearby intronic nucleotides can significantly impair splice site recognition and efficiency, as the donor splice site is defined not only by the invariant GT dinucleotide but also by adjacent sequences critical for U1 snRNP binding and spliceosome assembly [6,7]. Therefore, variants affecting positions such as +4 may weaken splice site strength, disrupt normal exon recognition, and lead to aberrant splicing.

In this case, the high SpliceAI score (0.80) and Pangolin score (0.72) predict a significant impact on normal splicing, which may result in exon skipping or activation of a nearby cryptic splice site, both mechanisms leading to the generation of an aberrant transcript that may disrupt the reading frame and introduce a premature termination codon, which in turn is expected to trigger nonsense-mediated mRNA decay (NMD) [7,17], resulting in reduced or absent production of functional PHEX protein and supporting a loss-of-function mechanism consistent with XLH, representing a plausible molecular hypothesis.

According to the American College of Medical Genetics and Genomics (ACMG) criteria, this variant meets the PP3 criterion, as computational splicing predictors support a deleterious effect, and the PM2 criterion, as it is absent or extremely rare in population databases with adequate coverage [18]. Although it is currently classified as a variant of uncertain significance (VUS), the integration of clinical, biochemical, radiological, genetic, and computational evidence supports its probable biological relevance and likely contribution to the observed phenotype.

Additionally, mild XLH phenotypes have been described in patients carrying variants located in noncoding regions of the PHEX gene, including intronic variants near splice sites (e.g., c.1900-4_1900-1dup), regulatory variants in the 3′-UTR (e.g., c.231A>G), and cases associated with mosaicism [19,20]. These observations expand the clinical and molecular spectrum of the disease and highlight the relevance of non-canonical variants in modulating disease expression.

In this context, although the patient exhibits clinical, biochemical, and radiological features consistent with XLH, the variant allele fraction of approximately 24% raises the possibility of somatic mosaicism, which may influence phenotypic expression. While the overall presentation is clearly compatible with XLH, the degree of severity may be modulated by the coexistence of wild-type and mutant cell populations, in line with the well-recognized clinical heterogeneity of the disorder [11,19].

Finally, this case highlights how the presence of parental consanguinity may bias the diagnostic approach toward autosomal recessive disorders, potentially delaying the recognition of X-linked conditions. This underscores the importance of maintaining a broad diagnostic perspective and critically integrating clinical, biochemical, and molecular findings, as well as considering non-canonical splicing variants as potentially pathogenic when supported by consistent phenotypic and computational evidence, even in the absence of functional validation.

## Conclusions

The presence of disproportionate postnatal short stature, genu varum, and hypophosphatemia should raise clinical suspicion of XLH. We report a noncanonical intronic PHEX variant (c.1768+4dup), not previously described in clinical databases or the literature, with a variant allele fraction of approximately 24%, suggesting low-level somatic mosaicism, a factor that may contribute to phenotypic variability and represents a diagnostic challenge. In silico analysis supports a splicing alteration that was not detected by clinical exome sequencing, thereby underscoring the diagnostic limitations of standard approaches. Accordingly, this case highlights the importance of expanding the diagnostic strategy through complementary molecular techniques in selected patients. Intronic variants located outside canonical splice sites may have clinically relevant effects and should be considered, particularly in patients with a strong phenotypic suspicion of monogenic disease. Therefore, integrating clinical findings with bioinformatic prediction tools, along with the careful selection of molecular testing and the use of complementary approaches when exome sequencing is inconclusive, is essential to reduce the risk of missed diagnoses and to further delineate the molecular spectrum of rare diseases.
